# Concurrent DNA hypomethylation and epigenomic reprogramming driven by androgen receptor binding in bladder cancer oncogenesis

**DOI:** 10.1002/ctm2.70153

**Published:** 2024-12-27

**Authors:** Yu Xiao, Lingao Ju, Wan Jin, Hongwei Peng, Zongning Zhou, Mengxue Yu, Zilin Xu, Gang Wang, Kaiyu Qian, Yi Zhang, Xinghuan Wang

**Affiliations:** ^1^ Department of Urology Zhongnan Hospital of Wuhan University Wuhan China; ^2^ Department of Biological Repositories Human Genetic Resources Preservation Center of Hubei Province Zhongnan Hospital of Wuhan University Wuhan China; ^3^ Hubei Key Laboratory of Urological Diseases Laboratory of Precision Medicine Zhongnan Hospital of Wuhan University Wuhan China; ^4^ Euler Technology ZGC Life Sciences Park Beijing China; ^5^ High Performance Computing Center the Peking‐Tsinghua College of Life Sciences Peking University Beijing China; ^6^ Wuhan Research Center for Infectious Diseases and Cancer Chinese Academy of Medical Sciences Wuhan China; ^7^ Medical Research Institute Frontier Science Center for Immunology and Metabolism Taikang Center for Life and Medical Sciences Wuhan University Wuhan China

1

The intratumor heterogeneity (ITH) of bladder cancer (BLCA) facilitates resistance to treatment and enables evasion of immune surveillance, directly impacting patient outcomes and clinical prognosis. Our group has newly developed a tool named EpiTrace, which enables tracking single‐cell (sc) evolution via chromatin accessibility^1^ and reveals the ITH of bladder cancer in all stages.^2^ We also found that the TM4SF1‐positive cancer subpopulation (TPCS) drives ITH diversification in BLCA via integrative single‐cell atlas analysis.^2^

Previous data suggest that Notch signalling might contribute to urothelium cell lineage specification.^2^ Compared with that in normal urothelium cells and other cancer cells, the RNA expression of HES1 and HES4, effectors of Notch signalling, at the single‐cell level is significantly lower in TPCS.^2^ As ASCL1/2 expression is difficult to assess via single‐cell RNA sequencing because of the low expression abundance resulting from the repression of the Notch signal, we used single‐cell assay for transposase‐accessible chromatin (scATAC) data to infer their transcriptional activity. Compared with those in other cells, scATAC‐inferred HES1 and HES4 expression levels were decreased in TPCS. In contrast, ASCL1 and ASCL2 transcription factors (TFs) activities are increased in TPCS without apparent transcriptional upregulation (Figure 1A), suggesting derepression of ASCL1/2 from HES1/4. In accordance with the results of the cell‐cell signalling analysis and coregulated transcription network activity, SMAD4 transcription activity was increased, whereas its RNA level did not change (Figure 1A). A change in transcription factor activity was also noted for other TFs, including FOXA1 (Figure 1A and Figures S1 and S2).

**FIGURE 1:**
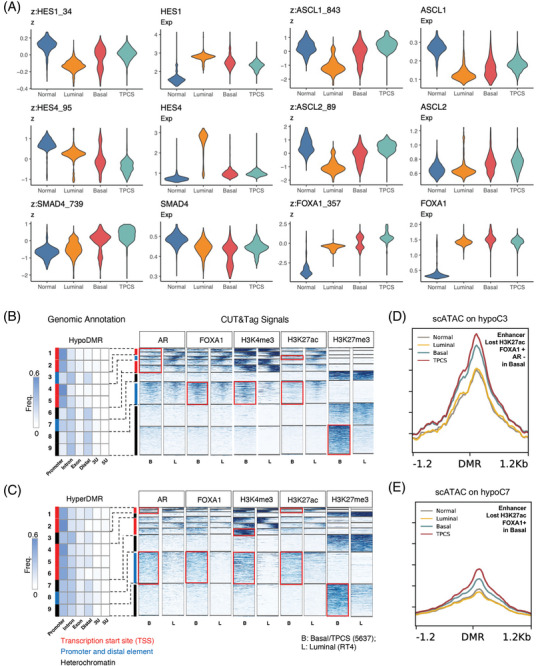
Androgen receptor (AR) binding reprogrammed enhancers epigenetically in TM4SF1‐positive cancer subpopulation (TPCS). (A) Transcription factor binding activity (z) and expression (Exp) of HES1, HES4, ASCL1, ASCL2, SMAD4 and FOXA1 in normal cells, luminal cancer cells, basal cancer cells, and TPCS from scATAC data. (B) Cleavage under target & tagmentation (CUT&Tag) profiling of AR‐binding, FOXA1‐binding, and H3K4me3, H3K27me3 and H3K27ac histone modifications in a basal cell line (5637, B) and a luminal cell line (RT4, L). Muscle‐invasive bladder cancer (MIBC)‐specific hypomethylated genomic loci are shown in the figure. Loci were K‐means clustered according to their epigenetic modification profiles into 10 clusters. Because the 10th cluster lacked any meaningful epigenetic modifications in this assay, their binding profile is not shown in the figure. Regulatory element annotations of the clusters are shown in the left panel. CUT&Tag signals are shown on the right panel. Cluster annotations according to their genomic location as well as histone modifications are shown in colored bars next to the CUT&Tag panel (labels at the bottom). The *HES4*, *GPX4* and *YWHAZ* genes are in the 7th cluster (hypoC7), which is a set of enhancers that are lost in basal cells. (C) CUT&Tag profiling of MIBC‐specific hypermethylated genomic loci. The labels are similar to those in B). (D) Single‐cell ATAC coverage of the hypoC3 cluster, which binds to FOXA1 in both basal and luminal cells but loses H3K27ac modification and AR binding in basal cells (red box in B). (E) Single‐cell ATAC coverage of the hypoC7 cluster, which lacks H3K27ac and is bound by FOXA1 in basal cells.

Elucidating the underlying molecular mechanisms for HES1 and HES4 repression requires the interrogation of the epigenetic information of both TPCS‐like and less malignant cancer cells. FOXA1 is known to be a master pioneering factor that is differentially expressed in muscle‐invasive bladder cancer (MIBC) downstream of BMP signalling and determines tumour phenotype.^3^ In prostate cancer, androgen receptor (AR) directly interacts with FOXA1, driving cancer growth and survival.^4^ BLCA incidence is 3–4 times higher in males^5^, ^6^; however, when diagnosed with BLCA, women tend to present with more advanced disease stages compared to men, resulting in poorer treatment outcomes and higher mortality rates.^7^, ^8^ Emerging research suggests that AR may facilitate the occurrence, progression, and recurrence of BLCA, potentially leading to the observed sex disparities.^9^ Therefore, we investigated how FOXA1 and AR binding epigenetically reprograms enhancers in BLCA epigenetically reprograms enhancers in BLCA.

We chose two cell lines for analysis: the RT4 cells with the TACC3‐FGFR3 fusion as a less malignant luminal‐like BLCA (Luminal, L) and the 5637 cells with homozygous TP53 mutation and amplified ERBB2 as an aggressive and malignant human muscle‐invasive BLCA (Basal, B) (Figures 1B,C and 2, and Dataset S1). Notably, as 5637 cells expressed high levels of KRT6A/B, we considered them basal‐like cell lines that mimic TPCS. By reanalyzing publicly available datasets and comparing them with our in‐house cleavage under target & tagmentation (CUT&Tag) data, we confirm that the quality of our CUT&Tag sequencing is equivalent to that of previously reported datasets (Figures S3–S5). We enriched known transcription factor‐binding site motifs in the RT4 AR peaks. As a control, we performed similar enrichment with the reference prostate cancer AR peaks (GSE70079). The enrichment results revealed that AR binding motifs were enriched in both datasets. However, the top enriched motif in both datasets was FOXA1 instead of AR, suggesting that AR binding to DNA is partially mediated by other transcription factors (Figure S6).

**FIGURE 2:**
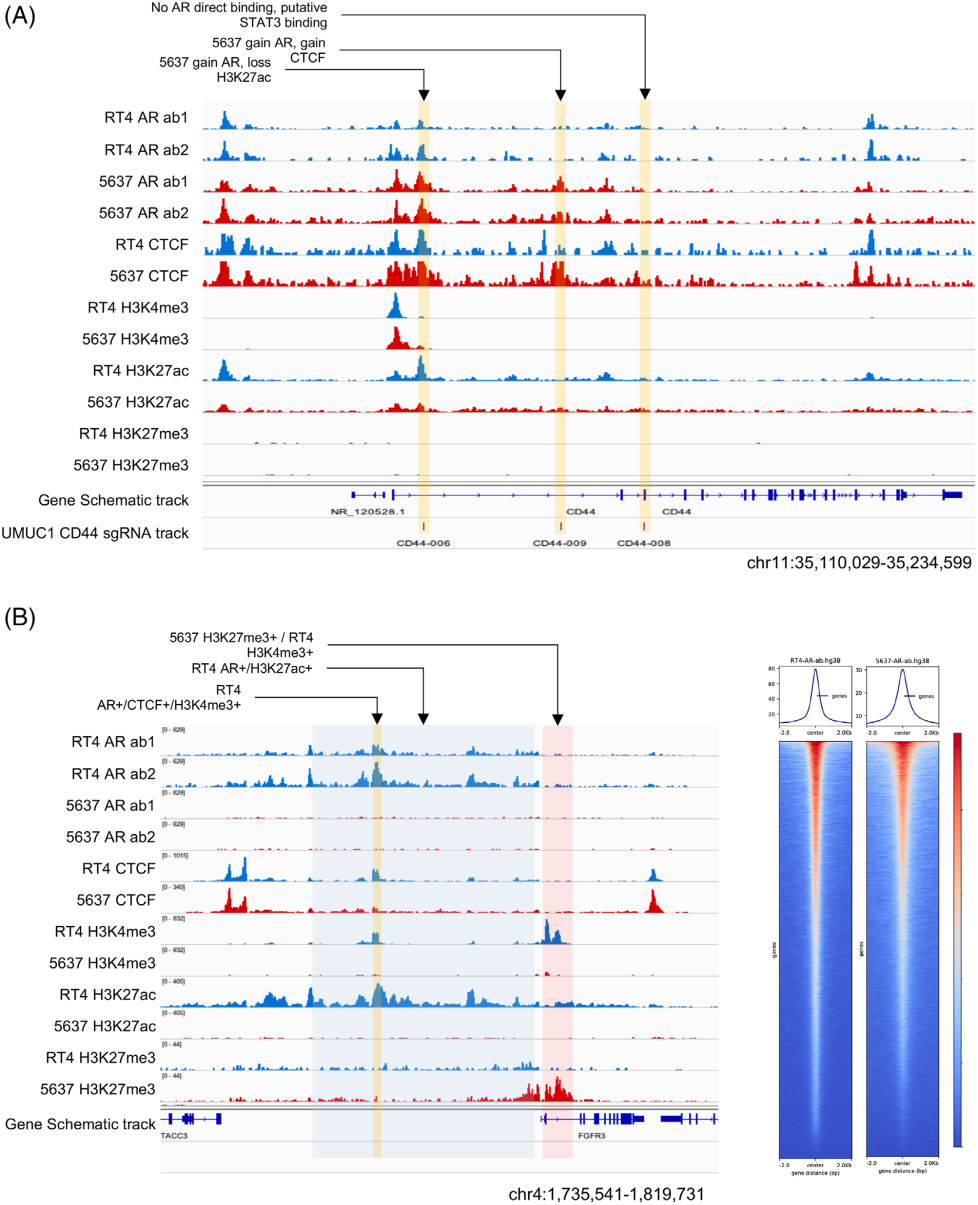
Differential epigenomic reprogramming in bladder cancer (BLCA) related to androgen receptor (AR). (A, B) Top‐to‐bottom: AR cleavage under target & tagmentation (CUT&Tag) in RT4 cells (two antibodies), AR CUT&Tag in 5637 cells (two antibodies), CTCF CUT&Tag in RT4 cells, CTCF CUT&Tag in 5637 cells, H3K4me3 CUT&Tag in RT4 cells, H3K4me3 CUT&Tag in 5637 cells, H3K27ac CUT&Tag in RT4 cells, H3K27ac CUT&Tag in 5637 cells, H3K27me3 CUT&Tag in RT4 cells and H3K27me3 CUT&Tag in 5637 cells. (A) Differential AR binding to the CD44 locus between RT4 and 5637 cells. (B) Differential AR binding to the TACC‐FGFR region in RT4 and 5637 cells. (C) AR binding profile across binding peaks in RT4 and 5637 cells.

In particular, the patterns of these transcription factors and histone modifications were used to demarcate different classes of chromatin states in tumour‐associated differentially methylated regions (DMRs) (hypoDMRs and hyperDMRs). These chromatin state classes were correlated with chromatin accessibility (ChrAcc) in single cancer cells derived from clinical samples. The hypoDMRs and hyperDMRs in MIBC were stratified into subgroups by utilizing profiles of histone modifications and transcription factor binding. These subgroups were annotated based on their histone modifications and genomic position information. MIBC‐specific hypoDMRs were divided into 10 clusters via k‐means clustering, with the first 9 clusters showing histone modifications on either AR, FOXA1, H3K4me3, H3K27ac or H3K27me3 (Figure 1B). The 10^th^ cluster showed no signal for any of these modifications and was not analyzed in this study. In TPCS, MIBC‐hypoDMR group 3 (hypoC3), an enhancer cluster, exhibited a decrease in AR binding, as well as H3K27ac modification (Figure 1B). MIBC‐hypoDMR group 7 (hypoC7), which is located proximal to several basal‐low genes, such as *GPX4* and *YWHAZ*, presented increased H3K4me3 and FOXA1 binding and decreased H3K27ac in TPCS (Figure 1B). The ChrAcc of single BLCA cells in hypoC3 and hypoC7 increased in basal cancer cells relative to luminal cancer cells and further increased in TPCS (Figure 1D,E).

In the hyperDMR sets, MIBC‐hyperDMR group 8 (hyperC8) comprised FOXA1‐bound, H3K27ac/H3K4me3‐high enhancers (Figure 1C). Moreover, MIBC‐hyperDMR group 6 (hyperC6) comprises promoters that are solely active in basal cancer cells (Figure 1C) and that regulate lineage specification genes, such as *HOXA3*/*HOXA5*, which are upregulated in normal Bs2/3 cells.^2^ These results suggest that DNA hypermethylation might not necessarily be transcriptionally suppressive. Similarly, we observed that DNA hypermethylation at the SOX2 locus is linked to its overexpression in basal cancer.^10^ Additionally, other loci that exhibited differential methylation in MIBC exhibited basal cancer‐specific H3K27me3 deposition, indicating regulation via PRC2‐dependent mechanisms.

Alterations in the epigenomic landscape of TPCS have a direct effect on the expression of genes. In T3 and T4a clinical samples enriched with TPCS, DNA demethylation in a region bound by FOXA1 and CTCF downstream of *HES4* was associated with the loss of long‐range chromatin interactions, loss of enhancer activity, and gain of repressive H3K27me3 marks around the HES4 locus (Figure S7A,C). Similarly, differential FOXA1 binding and *de novo* DNAm were associated with increased interaction between FOXA1‐bound active enhancers and the promoter of *TFAP2C* in TPCS (Figure S7B,D). Differential AR binding is also linked to lineage‐specific gene expression (Figure 2).

Taken together, our results suggest that during BLCA oncogenesis, AR binding reprogrammed DNA hypomethylation occurs concomitantly with epigenomic patterning to control specific transcription programs, which may drive the evolutionary heterogeneity of MIBC.

## AUTHOR CONTRIBUTIONS

Y.X., K.Q., Y.Z. and X.W. conceived and designed the study. Y.X., W.J., K.Q., and Y.Z. contributed to methodology. W.J. and Y.Z. performed computation and formal analysis. Y.X., L.J., W.J., G.W., K.Q. and Y.Z. performed the experiments and investigations. L.J., H.P., Z.Z., M.Y., Z.X., G.W., K.Q. and X.W. contributed to collecting and obtaining the study resources. Y.X., L.J., W.J. and Y.Z. wrote the manuscript. K.Q., Y.Z. and X.W. contributed to a critical review of the manuscript. All authors read and approved the final manuscript.

## CONFLICT OF INTEREST STATEMENT

The authors declared no conflict of interest.

## FUNDING INFORMATION

This study was supported by the National Natural Science Foundation of China (82472733), the Fundamental Research Funds for the Central Universities (2042022dx0003) and the Research Fund of Zhongnan Hospital of Wuhan University (YYXKNLJS2024001 and PTPP2024001).

## ETHICS STATEMENT

All research procedures were approved by the Institutional Review Board of the Zhongnan Hospital of Wuhan University (approval numbers: 2015029 and 2020102) and conducted in accordance with the Declaration of Helsinki. Informed consent was obtained from all subjects.

## Supporting information



Supporting Information

Supporting Information

## Data Availability

The sequencing data of human bladder cancer biospecimens used in this study were derived from our previous publication.^2^ The remaining data are available within the article or Supporting Information.
