# Supplementary material for: Concurrent DNA hypomethylation and epigenomic reprogramming driven by androgen receptor binding in bladder cancer oncogenesis
Source: Clin Transl Med. 2024 Dec 27;15(1):e70153. doi: 10.1002/ctm2.70153 (PMC11681005; doi:10.1002/ctm2.70153)
Supplement: Supplementary file 1 — Supporting Information [file CTM2-15-e70153-s002.pdf]

Supplementary Information

Concurrent DNA hypomethylation and epigenomic reprogramming driven by androgen receptor binding in bladder cancer oncogenesis

Materials and Methods: Pages 2-5

Supplementary Figures S1-S7: Pages 6-14

Description of Supplementary Dataset: Page 15

References for Supplementary Information: Page 16

Materials and Methods

Source sequencing data of human biospecimens

The sequencing data of the human biospecimens used in this paper were derived from our previous study.[1] The raw data and clinical information were uploaded to the Genome Sequence Archive for Human (*http://bigd.big.ac.cn/gsa-human/*) at the BIG Data Center, Beijing Institute of Genomics, Chinese Academy of Sciences (accession number: HRA001225). The raw sequencing data and clinical information are unique to an individual and require controlled access. The deposited and publicly available data are compliant with the regulations of the Cina Human Genetic Resources Management Office, Ministry of Science and Technology of China (approval number: 2022BAT0129). Due to local legal requirements, the in-house-generated human dataset will be available only in processed data format and will require a case-by-case application through the China Human Genetic Resources Management Office. The original code used during the study were provided at Github: https://github.com/MagpiePKU/Bladder_TPCS_Paper.

Cell lines

RT4 and 5637 cells were kindly provided by the Cell Bank, Chinese Academy of Sciences (Shanghai, China), and cultured under identical conditions following standard procedures.[2] To construct stable 5637-AR and RT4-AR cell lines, LV6-AR lentivirus packaging was provided by GenePharma Inc. (Shanghai, China). The lentivirus particles were used for infection of target cells with the supplement polybrene. After two rounds of infection, the cells were selected with 1 μg/mL puromycin.

Cleavage under target & tagmentation (CUT&Tag) sequencing of cells

RT4 or 5637 cells were counted via Trypan blue (Solarbio, Beijing, China). After quantification, 40 million cells were used for the CUT&Tag experiment. CUT&Tag experiments were performed with a NovoProtein CUT&Tag 2.0 pAG-Tn5 kit (NovoProtein, Cat. #N259) according to the manufacturer's protocol. The antibodies used in this study included anti-H3K4me3 (Diagenode, Cat. #C154100003), anti-H3K27me3 (Abcam, Cat. #ab6002), anti-H3K27ac (Abcam, Cat #ab4729), anti-FOXA1 (Abcam, Cat. #ab170933), anti-AR (Abcam, Cat. #ab108341), goat-anti-mouse IgG (Sangon, Cat. #D111024) and goat-anti-rabbit IgG (Sangon, Cat. #D111018) antibodies. Each library was sequenced to 2× human genome coverage on a NovaSeq sequencer (Illumina, CA, USA).

ATAC and CUT&Tag sequencing data preprocessing

All assay for transposase-accessible chromatin sequencing (ATAC-seq) data were processed exactly described in our previous study.[1] Raw paired-end open chromatin tagmentation (ATAC) and CUT&Tag sequencing data were mapped to the human reference genome GRCh38 via Bowtie2 (-k 10 --very-sensitive -X 2000) (*https://github.com/BenLangmead/bowtie2*). All unmapped reads, nonuniquely mapped reads, reads with low mapping quality (MAPQ < 20) and PCR duplicates were removed. For the CUT&Tag sequencing libraries, the following data were used. For in-house-prepared ATAC-seq data, the data were quality controlled by assessing the insertion size (using an in-house R script) and TSS enrichment (using an in-house R script with the GenomicRanges package (*https://github.com/Bioconductor/GenomicRanges*)) by measuring the depth ratio at the promoter region (GRCh38 refFlat annotation from the UCSC Genome Browser) (0 bp

of the TSS vs. 1 kbp +- of the TSS). A QC-passed ATAC-seq library must have a TSS enrichment of 6, mapped deduplicated sequencing fragments >= 20 M PE reads, PCB1>0.9, and PCB2>3 (*https://www.encodeproject.org/pipelines*). Enrichment peaks were determined by intersecting peaks found from MACS2 callpeak (-f BAMPE)[3] (*https://github.com/taoliu/MACS*) and Genrich (-r -m 1 -j; for ATAC only; and standard parameter for CUT&Tag) (*https://github.com/jsh58/Genrich*). Quality control of ATAC-seq libraries, including read length, V-plots and TSS enrichment, was performed with custom R scripts and deepTools (*https://github.com/deeptools/deepTools*). Reliable peaks were identified with IDR (*https://www.encodeproject.org/software/idr*). Reliable ATAC peaks from different sets of data were converged with a 1 bp minimum overlap and extended to the largest width of overlapping peaks. Combining these operations results in a set of nonoverlapping, varied-width peaks across the genome that encompass all reliable open chromatin regions.

Classification of differentially methylated regions by CUT&Tag signals

Reads from the CUT&Tag sequencing library were transformed into bigWigs via deepTools.[4] Windows +-1200 bp around the differentially methylated loci were used to extract the reads. The resulting coverage matrix containing H3K27me3, H3K27ac, H3K4me3, anti-FOXA1 and anti-AR signals was then clustered via K-means with deepTools.[4] Annotation of these K-means clustered regions was performed via ChIPseeker[5] using -1000 bp~500 bp as the promoter around the TSS, and their relative distributions in different types of genomic regions were plotted via pheatmap (*https://cran.r-project.org/web/packages/pheatmap/index.html*). Regions were labeled 'Promoters' if they were covered by H3K4me3 in any cell type, 'Enhancers' if they

were covered by H3K27ac in any cell type, and ‘Repressors’ if they were covered by H3K27me3 in any cell type. In these regions, we did not detect bivalent promoter/enhancer features with H3K4me3/H3K27me3 double positivity.

Supplementary Figures S1-S7

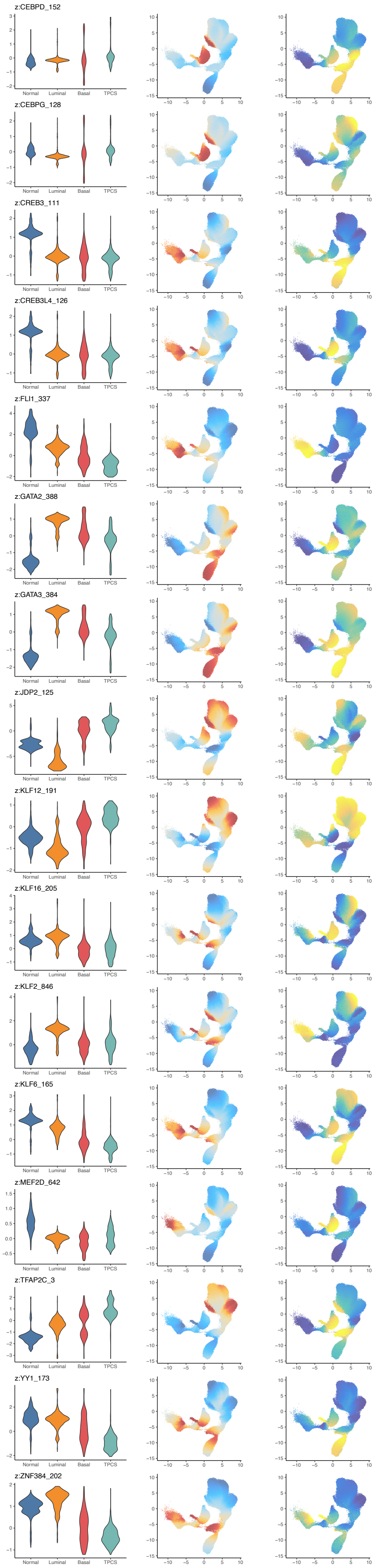

Supplementary Figure S1. Methylation sensitivity significantly altered TF activity in TPCS.

From left to right: **(Left)**: Z-normalized genome-wide transcription factor binding activity on a given TFBS motif measured in scATAC, grouped by classes of epithelial cells: normal, luminal (luminal-like cancer), basal (basal-like cancer), and TPCS (TM4SF1-positive cancer subpopulation). **(Middle)**: Z-normalized genome-wide transcription factor binding activity on a given TFBS motif measured in scATAC, projected to the UMAP manifold and smoothed by MAGIC in ArchR. **(Right)**: Deduced gene expression level of a given transcription factor in scATAC, projected to the UMAP manifold and smoothed by MAGIC in ArchR. The data shown in this figure are related to the following transcription factors: 1. Significant deviation in DNA-binding activity between TPCS and other types of epithelial cells; 2. Differential expression between TPCS and other types of epithelial cells; 3. The binding site is enriched in promoters of the TPCS-specific NMF metagene. 4. The binding site of (3) is reported to be methylation sensitive.[6]

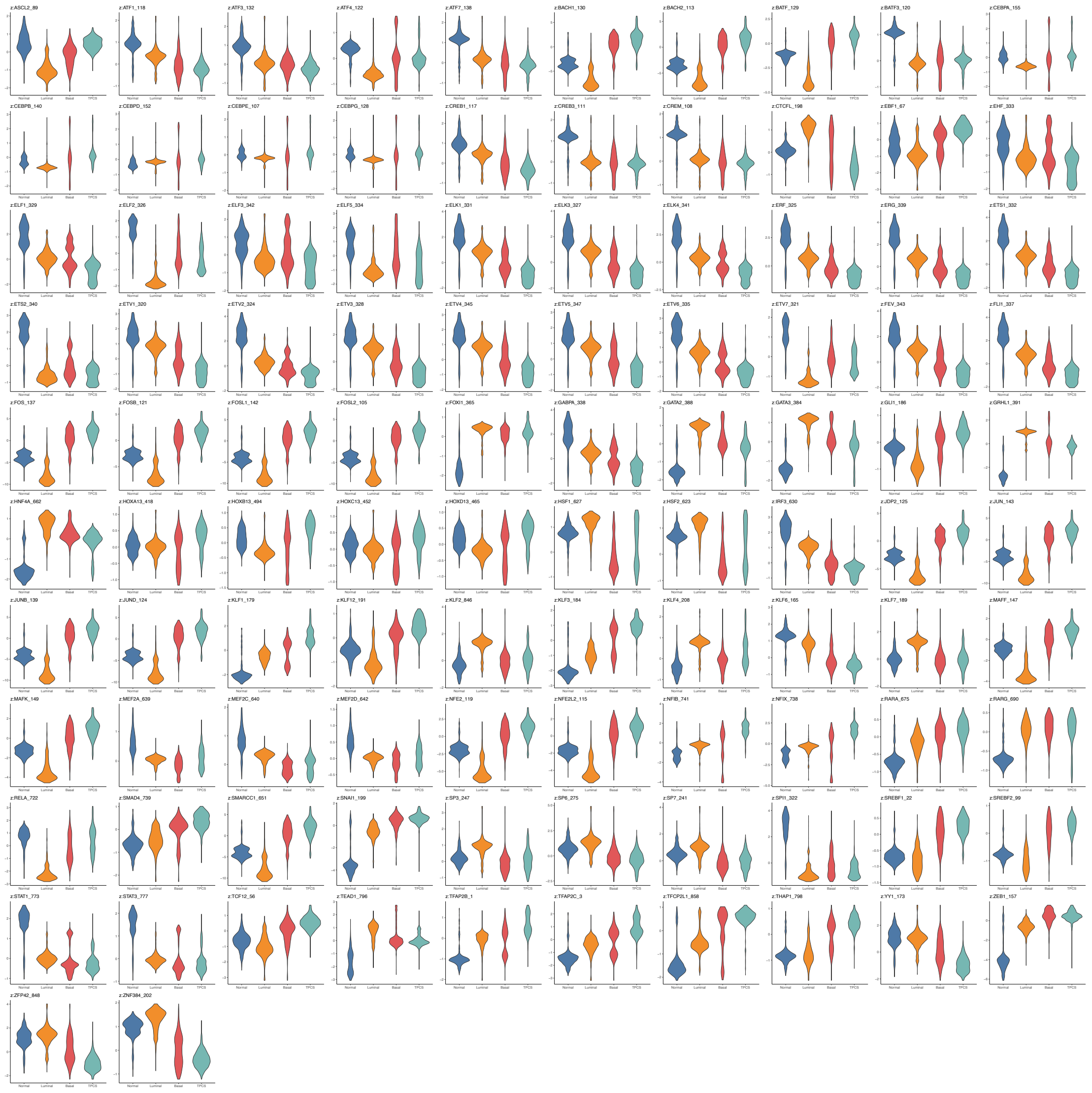

Supplementary Figure S2. Methylation insensitivity significantly altered TF activity in TPCS.

Each panel: Z-normalized genome-wide transcription factor binding activity on a given TFBS motif measured in scATAC, grouped by classes of epithelial cells: normal, luminal (luminal-like cancer), basal (basal-like cancer), and TPCS (TM4SF1-positive cancer subpopulation). The data shown in this figure are related to the following transcription factors: 1. Significant deviation in DNA-binding activity between TPCS and other types of epithelial cells; 2. The binding site is enriched in promoters of the TPCS-specific NMF metagene. 3. The binding site of (2) is unknown to be methylation sensitive.[6]

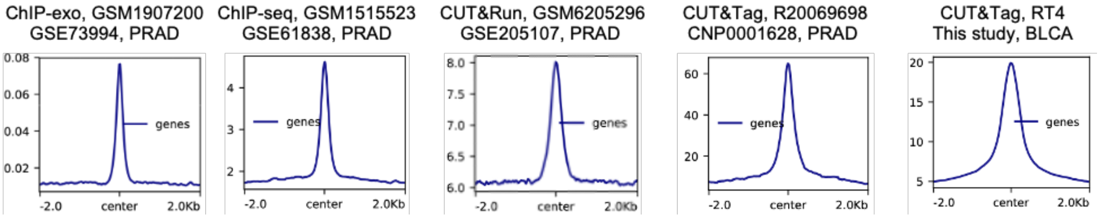

Supplementary Figure S3. Comparison of ChIP-exo, ChIP-seq, CUT&Run and CUT&Tag against known AR binding sites.

Data were downloaded from NCBI GEO and CNSA. Fastq files were processed similarly to the same pipeline used in the paper. Enrichments over reference AR peaks were performed via deepTools.

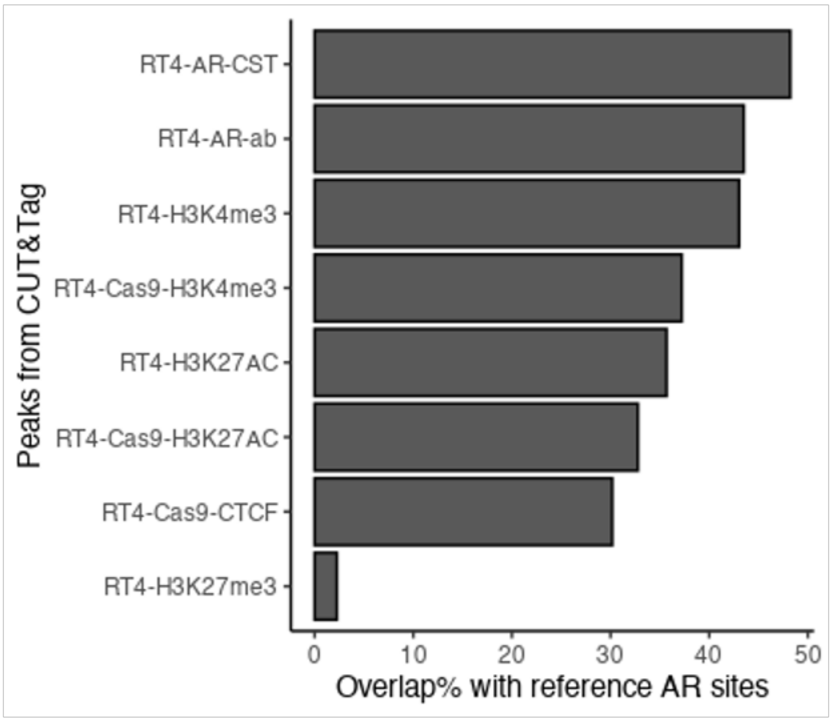

Supplementary Figure S4. Overlaps with reference AR binding sites.

The overlap fractions between the MACS2-called peaks and reference AR ChIP-seq peaks were computed in R (4.1.3).

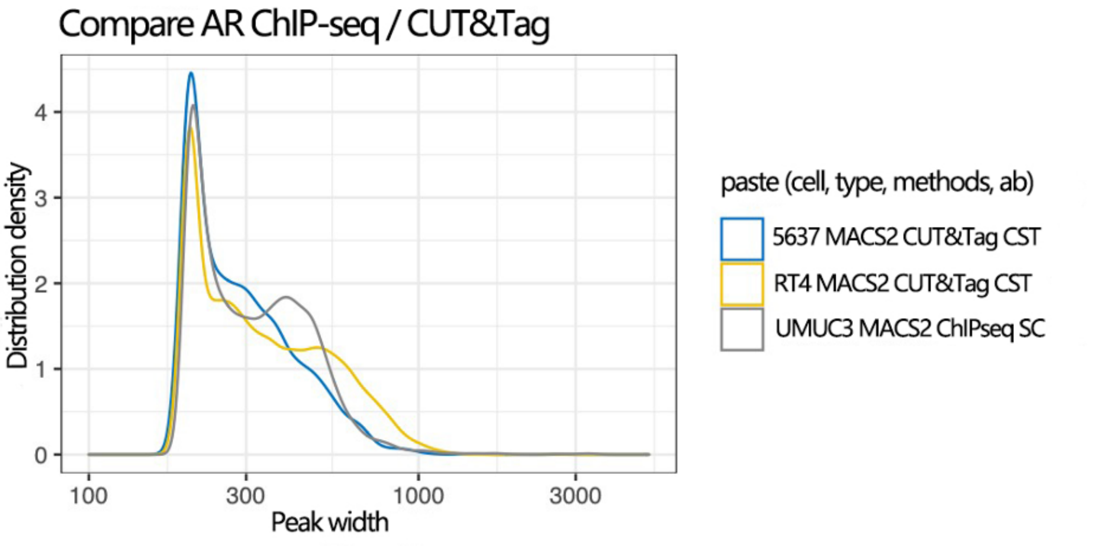

Supplementary Figure S5. Comparing AR ChIP-seq/CUT&Tag performance.

We downloaded the fastq files from the NCBI repository and reanalyzed the data in our pipeline. We compared peak width profiles from our AR CUT&Tag data and GSE147939. Notably, both the "reference" results and our results revealed many "peaks" covering long genomic loci. We found a few peaks with widths less than 100 bp. In general, we did not find significant differences between the reference dataset and the in-house datasets.

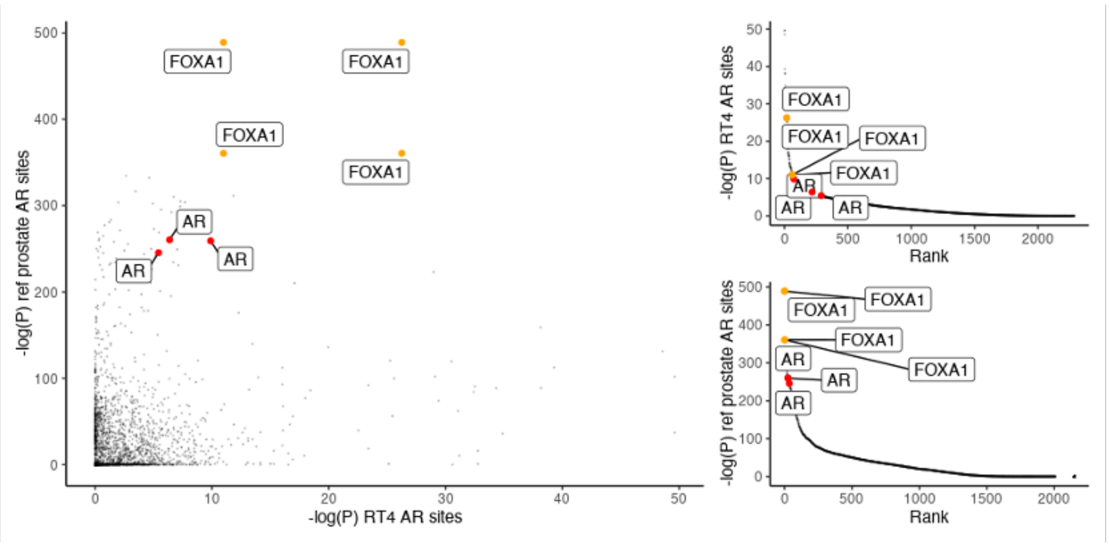

Supplementary Figure S6. Enrichment of known transcription factor-binding site motifs at AR sites.

Peaks were called via MACS2. Transcription factor-binding site enrichment was performed with PWMEnrich in R (4.1.3).

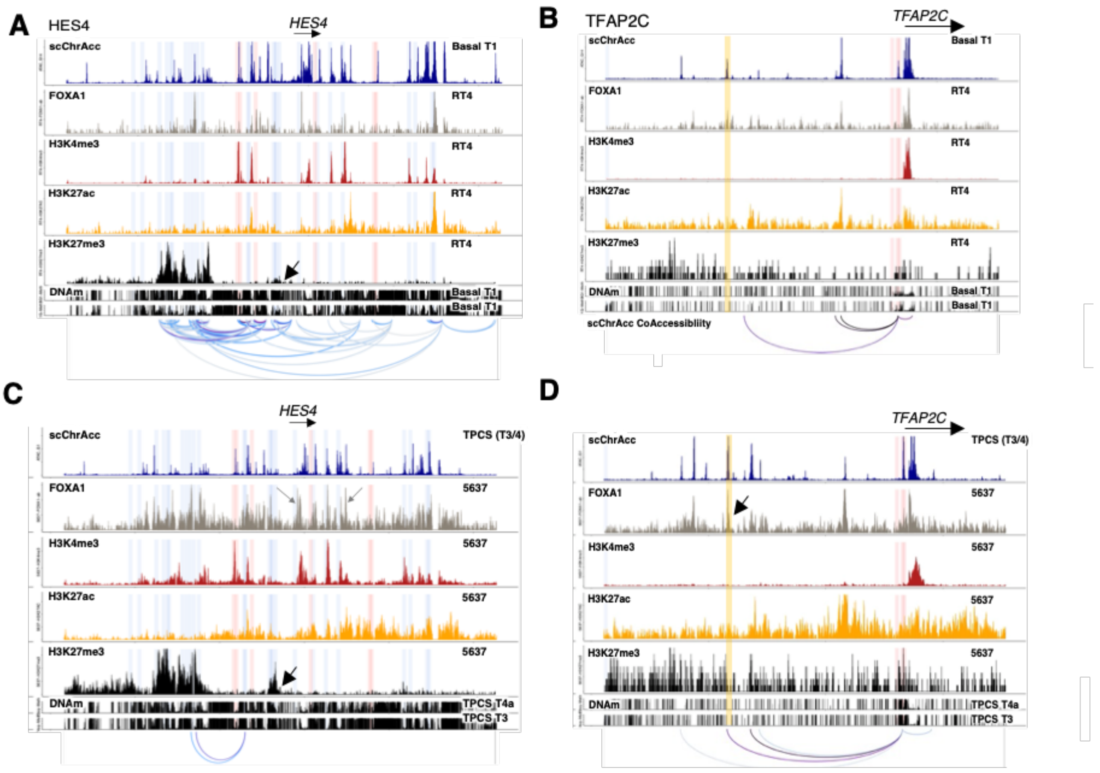

Supplementary Figure S7. DNA hypomethylation-associated epigenomic modification changes in BLCA.

Top to bottom tracks: single-cell chromatin accessibility (blue), FOXA1 CUT&Tag (gray), H3K4me3 (red), H3K27ac (orange), H3K27me3 (black), DNA methylation (black bars denoting 0--1 on each CpG), and single-cell chromatin co-accessibility (blue links). **(A)** HES4 locus epigenomic profile in early-stage BLCA cells (T1 basal cancer or RT4 cells); **(B)** TFAP2C locus epigenomic profile in early-stage BLCA cells; **(C)** HES4 locus epigenomic profile in late-stage BLCA cells (T3/4 TPCS or 5637 cells); **(D)** TFAP2C locus epigenomic profile in late-stage BLCA cells. The gene position is denoted by a horizontal arrow. In the HES4 locus of late-stage cancer cells, DNA hypomethylation (black arrow) coincides with H3K27me3 deposition on the HES4 promoter, resulting in loss of chromatin co-accessibility and reduced HES4 expression. In the TFAP2C locus in late-stage cancer cells, DNA hypermethylation of the promoter

results in increased co-accessibility between the promoter and a distal FOXA1-bound (black arrow) enhancer.

Description of Supplementary Dataset

Supplementary dataset S1. CUT&Tag peaks in RT4 and 5637 cells.

Reference genome is hg38. Differential Cut&Tag peaks between RT4 and 5637 were identified by macs2. “V1”: chromosome; “V2”: start; “V3”: end; “V4”: antibody information; “V5”: q-value.

References for Supplementary Information

[1] Xiao Y, Jin W, Qian K, et al. Integrative Single Cell Atlas Revealed Intratumoral Heterogeneity Generation from an Adaptive Epigenetic Cell State in Human Bladder Urothelial Carcinoma. *Adv Sci (Weinh)*. 2024;11(24):e2308438.

[2] Xiao Y, Ju L, Qian K, et al. Non-invasive diagnosis and surveillance of bladder cancer with driver and passenger DNA methylation in a prospective cohort study. *Clin Transl Med*. 2022;12(8):e1008.

[3] Zhang Y, Liu T, Meyer CA, et al. Model-based analysis of ChIP-Seq (MACS). *Genome Biol*. 2008;9(9):R137.

[4] Ramirez F, Dundar F, Diehl S, Gruning BA, Manke T. deepTools: a flexible platform for exploring deep-sequencing data. *Nucleic Acids Res*. 2014;42:W187-191.

[5] Yu G, Wang LG, He QY. ChIPseeker: an R/Bioconductor package for ChIP peak annotation, comparison and visualization. *Bioinformatics*. 2015;31(14):2382-2383.

[6] Yin Y, Morgunova E, Jolma A, et al. Impact of cytosine methylation on DNA binding specificities of human transcription factors. *Science*. 2017;356(6337):eaaj2239.
